# Silliman’s Journal

**Published:** 1829

**Authors:** 


36. *Silliman's Journal.* The distinguished Editor of the American Journal of Sciences, has lately made a powerful appeal to his countrymen, (or rather the enlightened portion of them) relative to the journal of which he is the projector and conductor. It is not his object to make it a source of pecuniary gain to himself, but of intellectual profit to the people among whom it circulates. Previously to the establishment of this most respectable periodical, not a few of the subjects which enrich its pages, were discussed in the journals of Medicine. The association was not unnatural, but in some respects inconvenient; and the Professor's design of instituting a magazine of Natural Philosophy, Chemistry, Mineralogy, Zoology, Botany and the other physical sciences, was creditable to himself, and in the execution may be made honorable to our country. It appears from the circular of which we have spoken, that the journal has never wanted able contributors, but that the patronage which it has received, has scarcely been sufficient to meet the expenses of publication. This ought not to have been the case, for such a work is imperiously wanted in America, and must not be suffered to languish. The subscription ought not only to equal the cost of publication, but afford the Editor a substantial reward.—In every pursuit the labourer is worthy of his hire, and in the case before us, the claim, which might be, but is not asserted, is one of the most valid that we have known. The Professor has devoted to his undertaking, a great deal of time in the most valuable period of his life, and the nation is reaping the fruits of his tireless exertions. The discoveries of our countrymen are made known, both at home and abroad, the scientific improvements of Europe are disseminated among us, a love for the useful arts and sciences is generated, their practical value is pointed out, genius is animated in its enterprizes, and patriotism supplied with the means of accomplishing its benificent ends. In all this our physicians are interested in common with other classes; but they have, or ought to have, other and more special motives for encouraging a journal of the physical sciences, from the enduring and intimate relations between those sciences and the profession of Medicine. To enumerate these connexions, would be a work of supererogation, every educated and reflecting medical man must perceive them; and admit, to himself at least, that it is his duty to contribute something towards the perpetuation of a work which lends so many aids to his own science, and promises to do so much for the character of his country.

